# Case Report: *TPR-ALK* fusion-positive inflammatory myofibroblastic tumour treated with sequential *ALK* inhibitors

**DOI:** 10.3389/fonc.2026.1778283

**Published:** 2026-03-05

**Authors:** Fabiano Flauto, Filippo Vitale, Caterina De Luca, Rosa Maria Di Crescenzo, Francesco Petteruti, Annarita Peddio, Giuseppe Neola, Vincenzo Damiano

**Affiliations:** 1 Department of Clinical Medicine and Surgery, University of Naples Federico II, Naples, Italy; 2 Department of Public Health, University of Naples Federico II, Naples, Italy; 3 Department of Advanced Biomedical Sciences, University of Naples, Naples, Italy; 4 U.O. Thoracic Surgery Pineta Grande Hospital, Castelvolturno, CE, Italy; 5 Portsmouth Hospitals University NHS Trust, Portsmouth, United Kingdom

**Keywords:** ALK-fusion, case report, inflammatory myofibroblastic tumour, lorlatinib, TPR-ALK

## Abstract

**Background:**

Inflammatory myofibroblastic tumour (IMT) is a rare mesenchymal neoplasm, frequently driven by oncogenic kinase fusions, most commonly involving anaplastic lymphoma kinase (*ALK*). Standard cytotoxic therapies have limited efficacy in unresectable IMT. *ALK* inhibitors such as crizotinib are recommended for *ALK*-positive IMT and achieve high response rates; however, disease progression may occur, and optimal management after crizotinib failure remains poorly defined.

**Case presentation:**

We report the case of a young adult woman with an *ALK*-rearranged thoracic IMT harbouring a rare *TPR-ALK* fusion. Initial treatment with crizotinib resulted in a partial radiologic response and rapid clinical improvement. After approximately three months of therapy, however, the disease progressed clinically and radiologically. Plasma-based DNA next-generation sequencing did not identify *ALK* resistance mutations or circulating fusion transcripts. The patient was subsequently treated with lorlatinib, a third-generation *ALK* inhibitor, which induced rapid and near-complete tumour regression. Treatment was well tolerated. The patient experienced marked improvement in performance status and quality of life and remains in ongoing radiologic response confirmed at 150 days of treatment.

**Conclusion:**

This case highlights the importance of comprehensive molecular testing in IMT to identify actionable *ALK* fusions and supports the use of sequential *ALK* inhibitor therapy. This *TPR-ALK* fusion-driven IMT demonstrates that disease progression after an initial response to crizotinib can be effectively overcome with lorlatinib, resulting in rapid and durable clinical benefit. These findings add to emerging evidence supporting next-generation *ALK* inhibitors as effective treatment options for *ALK*-rearranged IMT after crizotinib failure.

## 1 Introduction

Inflammatory myofibroblastic tumour (IMT) is a rare mesenchymal neoplasm classified as an intermediate-grade sarcoma (1). It most often occurs in children and young adults and can arise in diverse anatomical locations, commonly the lung and abdominopelvic cavity (1). Histologically, IMT is characterized by myofibroblastic spindle cells accompanied by a prominent inflammatory infiltrate (2). While surgical resection is curative for localized disease, advanced or unresectable IMTs pose a therapeutic challenge (1, 2). Traditional chemotherapy and radiation have shown limited benefit, as IMTs are generally resistant to conventional therapies (3). A defining feature in a majority of IMTs is the presence of oncogenic gene fusions, most notably involving the anaplastic lymphoma kinase (*ALK*) gene (4). *ALK* rearrangements have been identified in approximately 50-80% of IMTs, leading to aberrant *ALK* tyrosine kinase activity that drives tumour growth. These *ALK* fusions include various partner genes such as *TPM3*, *TPM4*, *CLTC*, *RANBP2* and confer sensitivity to *ALK*-targeted therapies (2, 4). The discovery of *ALK* fusions in IMT has rapidly influenced treatment strategies. Based on case reports and clinical trials, crizotinib, a first-generation *ALK* inhibitor, has been incorporated as a recommended systemic therapy for *ALK*-positive IMT (5). In a phase II trial of advanced *ALK*-positive IMTs, crizotinib achieved an objective response rate of nearly 50%, including complete responses, with disease control in all patients (6).

Despite these therapeutic advances, resistance to *ALK* inhibitors can develop, as observed in other *ALK*-driven malignancies (7). In *ALK*-rearranged non-small cell lung cancer (NSCLC), the sequential administration of next-generation *ALK* inhibitors following crizotinib failure has led to significant improvements in clinical outcomes by overcoming resistance-associated mutations (8). In contrast, resistance mechanisms in *ALK*-rearranged IMT and the clinical activity of newer-generation *ALK* inhibitors in this disease remain poorly characterized. Documented mechanisms of acquired resistance in IMT include secondary mutations within the *ALK* kinase domain, such as the *ALK*^G1269A^ mutation identified after prolonged crizotinib exposure in a reported case (9). Consequently, improving our understanding of therapeutic strategies for *ALK*-positive IMTs that progress on first-line treatment remains an important unmet need.

Here we present a unique case of a young adult patient with an *ALK*-positive thoracic IMT driven by a rare *TPR-ALK* fusion. The *TPR-ALK* fusion oncoprotein has been described in other malignancies, including NSCLC and anaplastic large-cell lymphoma (10). The case highlights the importance of comprehensive molecular diagnostics in rare tumours and demonstrates how precision oncology can yield dramatic, life-altering results even after initial therapy failure. It also illustrates a successful strategy to overcome *ALK* inhibitor resistance in IMT, contributing valuable insights to the limited literature on sequential *ALK*-targeted therapy in this rare disease. This case report is written in accordance with the CARE guidelines, and the patient provided informed consent for publication.

## 2 Case presentation

A young adult woman in her late twenties, with no relevant past medical conditions, family history of cancer, or psychosocial comorbidities, presented with a several-month history of persistent dry cough, intermittent low-grade fever, progressive chest pain, and bilateral ankle oedema. No relevant occupational exposures or psychosocial stressors were reported. Initial laboratory tests revealed elevated inflammatory markers. Early chest imaging identified a small, well-circumscribed lesion in the left lower lobe, which was initially interpreted as a benign cystic lesion and managed conservatively.

**Figure 1:**
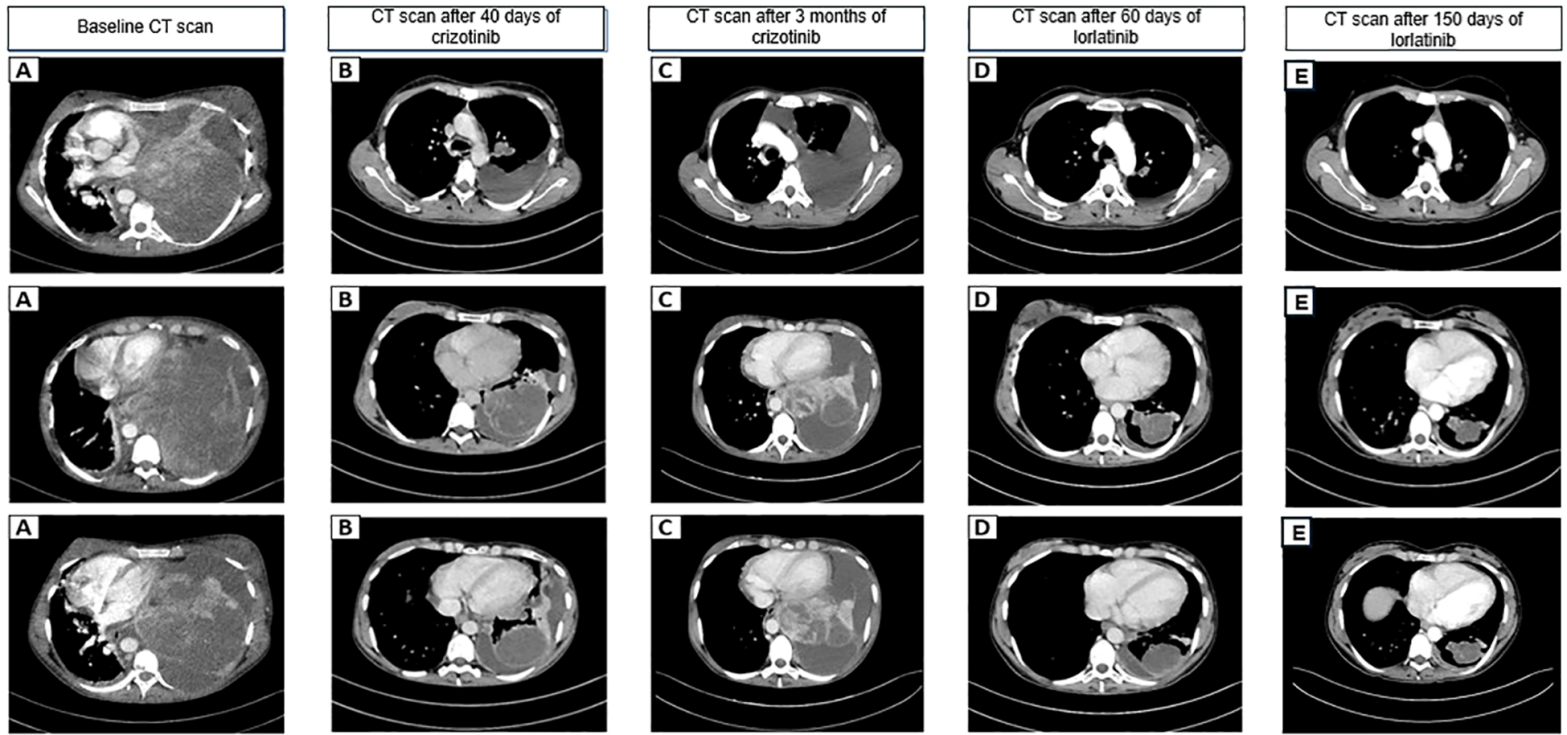
Radiologic timeline of disease evolution and treatment response. **(A)** Baseline: large heterogeneous mass occupying the left hemithorax with complete lung collapse and mediastinal shift. **(B)** After 40 days of crizotinib: partial response (PR) with reduction in the longest tumour diameter (-48% vs baseline) and reduction of mass effect. **(C)** After approximately 3 months of crizotinib: progressive disease (PD) with increase in the longest tumour diameter (+32% vs nadir) and renewed mediastinal compression. **(D)** After 60 days of lorlatinib: partial response (PR) with marked reduction in tumour size (-68% vs baseline; -74% vs progression) and re-expansion of the left lung. **(E)** After 150 days of lorlatinib: maintained radiologic response with no evidence of disease progression, confirming sustained disease control beyond the initial 3-month treatment period.

Over the following months, the patient experienced progressive clinical deterioration, with worsening respiratory symptoms. At referral to our tertiary care centre, she presented with an Eastern Cooperative Oncology Group (ECOG) performance status of 2, required continuous supplemental oxygen, and showed signs suggestive of impending mediastinal syndrome, including facial swelling and jugular venous distension. Contrast-enhanced computed tomography (CT) demonstrated a large heterogeneous mass measuring approximately 34×18 cm, occupying the left hemithorax, causing complete collapse of the left lung, mediastinal shift, and pericardial compression (**Figure 1A**).

Core needle biopsy and endobronchial sampling revealed a spindle cell neoplasm embedded in a myxoid stroma with a prominent lymphoplasmacytic inflammatory infiltrate. Immunohistochemical analysis showed diffuse vimentin positivity and CD34 expression in tumour cells, with CD45 highlighting the inflammatory component and a high Ki-67 proliferative index. Based on these findings, a vascular or lymphoproliferative process was initially suspected. Concomitantly, the presence of fungal elements in tissue samples and elevated serum galactomannan levels led to the initiation of empirical antifungal therapy under close multidisciplinary supervision.

**Figure 2:**
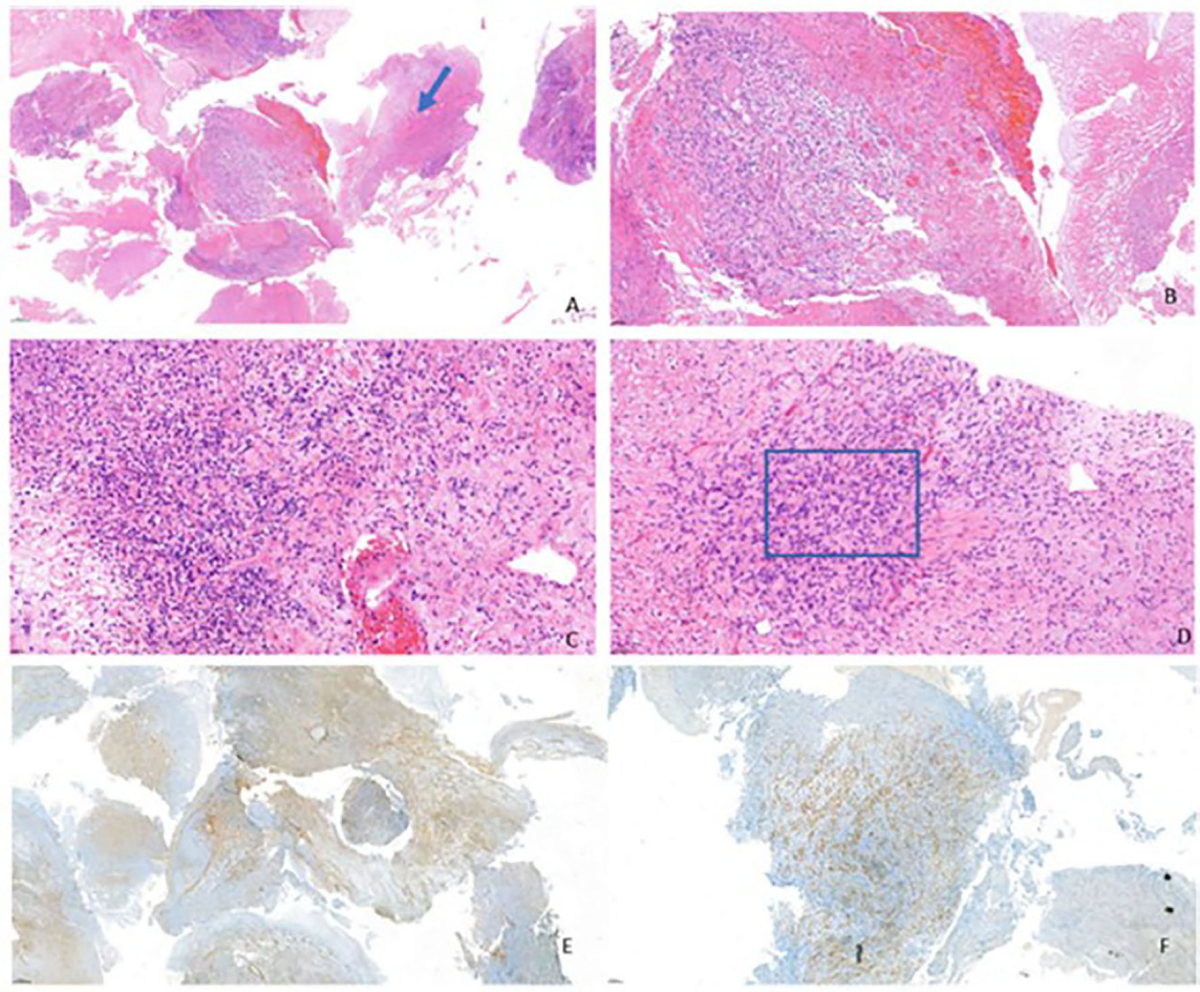
The histological examination revealed a high-grade malignant tumour with massive necrosis **(A)** and bundles of malignant spindle cells **(B)** associated with inflammatory infiltrate in which there were some eosinophils **(C, D)**. At Immunohistochemistry, these cells were positive for Actin **(E)** and *ALK***(F)**, suggesting the diagnosis of inflammatory myofibroblastic tumour.

**Figure 3:**
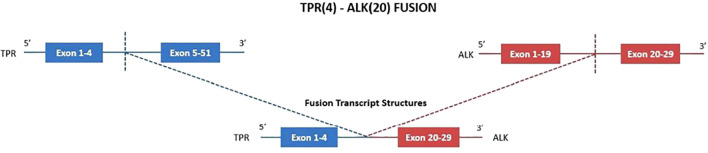
*TPR-ALK* fusion detected on RNA extracted from biopsy tissue by Oncomine Precision Assay GX on Genexus platform. The gene fusion generates a transcript with a break point at 5’ in exon 4 of *TPR* gene and at 3’ in exon 20 of *ALK* gene. .

Despite treatment, the patient experienced continued clinical and radiologic deterioration, and no microbiological confirmation of fungal infection was ultimately obtained. Following expert pathology review, the diagnosis was revised to IMT, supported by diffuse *ALK* immunoreactivity and focal positivity for smooth muscle actin and desmin (**Figure 2**). RNA-based next-generation sequencing (NGS) performed on tumour tissue identified a *TPR-ALK* gene fusion (**Figure 3**). Baseline plasma-based NGS, using a DNA-based assay, did not detect circulating tumour DNA alterations.

Given the identification of an *ALK* rearrangement, first-line targeted therapy with crizotinib 250 mg twice daily was initiated. Treatment was well tolerated and resulted in rapid clinical improvement, with complete resolution of respiratory symptoms and recovery of functional status within three weeks. Restaging CT performed approximately forty days after treatment initiation demonstrated a partial radiologic response, with nearly 50% reduction in tumour volume and near-complete restoration of thoracic anatomy (**Figure 1B**). Crizotinib was overall well tolerated. No dose interruptions or reductions were required.

After approximately three months of therapy, the patient developed recurrent cough, dyspnoea at rest, fever, and tachycardia. Repeat plasma-based DNA NGS again failed to identify *ALK* resistance mutations or other actionable alterations. CT imaging confirmed radiologic disease progression according to RECIST v1.1 criteria (**Figure 1C**). Due to the patient’s rapidly progressive clinical condition and critical respiratory compromise, repeat tissue biopsy was not pursued, as invasive diagnostic procedures were considered unsafe at that time.

Based on the aggressive clinical course and available evidence supporting the activity of next-generation *ALK* inhibitors after crizotinib failure, off-label treatment with lorlatinib 100 mg once daily was initiated. During treatment, the patient was regularly monitored for potential lorlatinib-related metabolic adverse events. Serial laboratory assessments did not reveal clinically significant hypercholesterolemia or hypertriglyceridemia. No new-onset hypertension, hyperglycaemia, or relevant weight gain were observed. No lipid-lowering, antihypertensive, or antidiabetic therapy was required. Follow-up CT imaging after approximately two months demonstrated marked tumour regression with full re-expansion of the left lung (**Figure 1D**), accompanied by complete resolution of systemic and respiratory symptoms. Follow-up CT imaging performed at 150 days from the start of lorlatinib confirmed maintained radiologic response with no evidence of disease progression. Treatment is ongoing, and the patient has now received therapy for more than three months, with sustained clinical improvement. A comprehensive timeline summarizing diagnostic assessments, therapeutic interventions, and outcomes is provided in **Figure 1**.

## 3 Discussion

This case highlights several clinically and methodologically relevant aspects in the management of IMT, particularly in the setting of rare ALK fusion variants and therapeutic sequencing. We report a thoracic IMT driven by a rare *TPR-ALK* fusion, characterized by an initial response to crizotinib followed by disease progression and a subsequent rapid and durable response to lorlatinib.

RNA-based sequencing identified a rare *TPR-ALK* fusion not previously reported in IMT, characterized by a 5′ breakpoint in exon 4 of *TPR* and a 3′ breakpoint in exon 20 of *ALK*. *TPR* encodes a large coiled-coil protein of the nuclear pore complex involved in nucleocytoplasmic transport and mRNA export. Importantly, retention of the TPR coiled-coil domain mediates constitutive dimerization, a structural feature known to drive ligand-independent kinase activation when fused to receptor tyrosine kinases. Similar oncogenic mechanisms have been reported for *TPR-MET* and *TPR-NTRK1* chimeras, as well as for *TPR-ALK* fusions previously described in NSCLC (9–11). The identification of this rearrangement was a critical component of the diagnostic work-up and underscores the diagnostic value of RNA-based NGS on tissue samples in detecting cryptic or atypical fusions that may be missed by DNA-based NGS or conventional assays such as fluorescence *in situ* hybridization (12).

DNA-based NGS assays rely on adequate intronic coverage and may have reduced sensitivity for detecting rare or non-canonical gene rearrangements, particularly for genes such as *ALK*, which are characterized by large and complex intronic regions. As a result, rare fusion partners and atypical breakpoint configurations may be missed by DNA-based approaches, both in tissue samples and in plasma-based assays (13, 14).

In selected NSCLC series, detection rates for *ALK* gene fusions using DNA-based assays have been reported to range from approximately 60-70% in treatment-naïve patients and from 40-50% in patients with disease progression after tyrosine kinase inhibitor (TKI) therapy (15). The specificity of these assays has been reported to range between approximately 90% and 100% (16). Although these data derive from a different disease context, they further support the concept that DNA-based approaches may have limited sensitivity for detecting certain fusion events.

In this context, repeated negative liquid biopsy results obtained using DNA-based NGS may be explained not only by biological factors, such as the predominantly localized growth pattern of IMT and the consequent low circulating tumour DNA shedding, but also by intrinsic technical limitations of DNA-based fusion detection (17). Accordingly, such results should be interpreted with caution and do not exclude the presence of an oncogenic fusion or ongoing *ALK* signalling when tissue-based molecular data are available.

Crizotinib has been widely adopted as a first-line agent for *ALK*-positive IMT, demonstrating meaningful clinical activity across multiple studies (5, 6, 18, 19). In the literature, cases of disease progression both during treatment and after treatment discontinuation with crizotinib have been reported (20, 21). In the present case, the patient achieved a partial radiologic response as the best response to crizotinib, accompanied by rapid clinical improvement, followed by disease progression after approximately three months of therapy.

This pattern is consistent with acquired resistance, given the initial response to first-generation *ALK* inhibition (22). In the absence of repeat tissue sampling at progression, the biological mechanisms underlying this early resistance remain speculative. Potential explanations include *ALK*-dependent on-target resistance, within the *ALK* tyrosine kinase domain, which induce resistance by way of direct steric hindrance of TKI binding, alteration in protein kinase conformation, and/or changes in ATP binding (23–25). Importantly, the marked and rapid response to subsequent lorlatinib therapy suggests persistent *ALK* dependency rather than complete pathway escape.

From a treatment-sequencing perspective, no standardized approach exists following crizotinib failure in IMT (1). Emerging evidence supports the activity of next-generation *ALK* inhibitors in *ALK*-rearranged IMT after crizotinib failure although available data remain limited to case reports and small series (26). While second-generation *ALK* inhibitors such as ceritinib or alectinib have demonstrated activity in isolated reports, the selection of lorlatinib in this case was guided by the aggressive clinical course and by its broader inhibitory spectrum and higher potency against *ALK* (8, 10).

This case has inherent limitations. Repeat tissue biopsy at progression was not feasible due to the patient’s critical clinical condition, precluding direct molecular characterization of resistance mechanisms. Moreover, as a single case report, these findings cannot be generalized.

Overall, this case supports a personalized and adaptive treatment approach in *ALK*-rearranged IMT. The identification of rare fusion partners through comprehensive genomic profiling can directly inform therapeutic decisions, and the observed efficacy of lorlatinib after crizotinib failure supports the feasibility of sequential *ALK* inhibition. As additional cases are reported and clinical experience accumulates, these insights may help refine diagnostic strategies and therapeutic sequencing in this rare and molecularly heterogeneous disease.

## 4 Patient perspective

The patient reported a dramatic improvement in quality of life after starting lorlatinib, with resolution of respiratory symptoms and return to normal daily activities. She expressed relief after months of uncertainty and emphasized the importance of molecular diagnosis in guiding effective treatment.

## 5 Conclusion

This case illustrates the clinical value of precision oncology in managing *ALK*-rearranged IMT. The identification of a rare *TPR-ALK* fusion enabled a targeted therapeutic approach, with an initial response to crizotinib, consistent with established *ALK* dependence in IMT, followed by rapid and durable disease control maintained beyond 150 days of treatment with lorlatinib. This outcome reinforces the importance of comprehensive molecular diagnostics and supports the rationale for sequential *ALK* inhibitor therapy in IMT. The sustained response suggests that long-term *ALK* inhibition may effectively control disease, raising questions about treatment duration and the potential integration of surgery in select cases. As more data emerge from real-world experiences, treatment paradigms for *ALK-*rearranged IMT will continue to evolve, aiming to optimize outcomes in this rare but increasingly treatable condition.

## Data Availability

The original contributions presented in the study are included in the article/supplementary material. Further inquiries can be directed to the corresponding authors.

## References

[1] von Mehren M Kane JM Agulnik M Bui MM Carr-Ascher J Choy E . Soft tissue sarcoma, version 2.2022, NCCN clinical practice guidelines in oncology. J Natl Compr Canc Netw. (2022) 20:815–33. doi: 10.6004/jnccn.2022.0035, PMID: 35830886 PMC10186762

[2] Gros L Dei Tos AP Jones RL Digklia A . Inflammatory myofibroblastic tumour: state of the art. Cancers (Basel). (2022) 14:3662. doi: 10.3390/cancers14153662, PMID: 35954326 PMC9367282

[3] Baldi GG Brahmi M Lo Vullo S Cojocaru E Mir O Casanova M . The activity of chemotherapy in inflammatory myofibroblastic tumors: A multicenter, european retrospective case series analysis. Oncologist. (2020) 25:e1777–84. doi: 10.1634/theoncologist.2020-0352, PMID: 32584482 PMC7648357

[4] Lovly CM Gupta A Lipson D Otto G Brennan T Chung CT . Inflammatory myofibroblastic tumors harbor multiple potentially actionable kinase fusions. Cancer Discov. (2014) 4:889–95. doi: 10.1158/2159-8290.CD-14-0377, PMID: 24875859 PMC4125481

[5] Theilen TM Soerensen J Bochennek K Becker M Schwabe D Rolle U . Crizotinib in ALK+ inflammatory myofibroblastic tumors-Current experience and future perspectives. Pediatr Blood Cancer. (2018) 65. doi: 10.1002/pbc.26920, PMID: 29286567

[6] Schöffski P Kubickova M Wozniak A Blay JY Strauss SJ Stacchiotti S . Long-term efficacy update of crizotinib in patients with advanced, inoperable inflammatory myofibroblastic tumour from EORTC trial 90101 CREATE. Eur J Cancer. (2021) 156:12–23. doi: 10.1016/j.ejca.2021.07.016, PMID: 34392187

[7] Cao Z Gao Q Fu M Ni N Pei Y Ou WB . Anaplastic lymphoma kinase fusions: Roles in cancer and therapeutic perspectives. Oncol Lett. (2019) 17:2020–30. doi: 10.3892/ol.2018.9856, PMID: 30675269 PMC6341817

[8] Wang QA Chen HW Wu RC Wu CE . Update of diagnosis and targeted therapy for ALK+ Inflammation myofibroblastic tumor. Curr Treat Options Oncol. (2023) 24:1683–702. doi: 10.1007/s11864-023-01144-6, PMID: 37938503 PMC10781869

[9] Xu X Li H Peng K Yu Y Chen L Fang Y . ALK-G1269A mutation in epithelioid inflammatory myofibroblastic sarcoma after progression on crizotinib: A case report. Oncol Lett. (2019) 17:2370–6. doi: 10.3892/ol.2018.9865, PMID: 30675302 PMC6341694

[10] Yamamoto H Yoshida A Taguchi K Kohashi K Hatanaka Y Yamashita A . ROS1 and NTRK3 gene rearrangements in inflammatory myofibroblastic tumours. Histopathology. (2016) 69:72–83. doi: 10.1111/his.12910, PMID: 26647767

[11] Choi YL Lira ME Hong M Kim RN Choi SJ Song JY . A novel fusion of TPR and ALK in lung adenocarcinoma. J Thorac Oncol. (2014) 9:563–6. doi: 10.1097/JTO.0000000000000093, PMID: 24736082

[12] Benayed R Offin M Mullaney K Sukhadia P Rios K Desmeules P . High yield of RNA sequencing for targetable kinase fusions in lung adenocarcinomas with no mitogenic driver alteration detected by DNA sequencing and low tumor mutation burden. Clin Cancer Res. (2019) 25:4712–22. doi: 10.1158/1078-0432.CCR-19-0225, PMID: 31028088 PMC6679790

[13] Heydt C Wölwer CB Velazquez Camacho O Wagener-Ryczek S Pappesch R Siemanowski J . Detection of gene fusions using targeted next-generation sequencing: a comparative evaluation. BMC Med Genomics. (2021) 14:62. doi: 10.1186/s12920-021-00909-y, PMID: 33639937 PMC7912891

[14] Jennings LJ Arcila ME Corless C Kamel-Reid S Lubin IM Pfeifer J . Guidelines for validation of next-generation sequencing-based oncology panels: A joint consensus recommendation of the association for molecular pathology and college of american pathologists. J Mol Diagn. (2017) 19:341–65. doi: 10.1016/j.jmoldx.2017.01.011, PMID: 28341590 PMC6941185

[15] Mezquita L Swalduz A Jovelet C Ortiz-Cuaran S Howarth K Planchard D . Clinical relevance of an amplicon-based liquid biopsy for detecting ALK and *ROS1* fusion and resistance mutations in patients with non-small-cell lung cancer. JCO Precis Oncol. (2020). doi: 10.1200/PO.19.00281, PMID: 32923908 PMC7448797

[16] Schram AM Chang MT Jonsson P Drilon A . Fusions in solid tumours: diagnostic strategies, targeted therapy, and acquired resistance. Nat Rev Clin Oncol. (2017) 14:735–48. doi: 10.1038/nrclinonc.2017.127, PMID: 28857077 PMC10452928

[17] Pascual J Attard G Bidard FC Curigliano G De Mattos-Arruda L Diehn M . ESMO recommendations on the use of circulating tumour DNA assays for patients with cancer: a report from the ESMO Precision Medicine Working Group. Ann Oncol. (2022) 33:750–68. doi: 10.1016/j.annonc.2022.05.520, PMID: 35809752

[18] Butrynski JE D'Adamo DR Hornick JL Dal Cin P Antonescu CR Jhanwar SC . Crizotinib in ALK-rearranged inflammatory myofibroblastic tumor. N Engl J Med. (2010) 363:1727–33. doi: 10.1056/NEJMoa1007056, PMID: 20979472 PMC3014292

[19] Gambacorti-Passerini C Orlov S Zhang L Braiteh F Huang H Esaki T . Long-term effects of crizotinib in ALK-positive tumors (excluding NSCLC): A phase 1b open-label study. Am J Hematol. (2018) 93:607–14. doi: 10.1002/ajh.25043, PMID: 29352732 PMC5947833

[20] Yıldırım ÜM Kebudi R Zülfikar B Bilgiç B . Inflammatory myofibroblastic tumors in children: clinical characteristics and treatment outcomes with a focus on targeted therapies. Turk J Pediatr. (2025) 67:51–60. doi: 10.24953/turkjpediatr.2025.5463, PMID: 40084721

[21] Michels SYF Scheel AH Wündisch T Heuckmann JM Menon R Puesken M . *ALK*^G1269A^ mutation as a potential mechanism of acquired resistance to crizotinib in an *ALK*-rearranged inflammatory myofibroblastic tumor. NPJ Precis Oncol. (2017) 1:4. doi: 10.1038/s41698-017-0004-3, PMID: 29872693 PMC5871789

[22] Poei D Ali S Ye S Hsu R . ALK inhibitors in cancer: mechanisms of resistance and therapeutic management strategies. Cancer Drug Resist. (2024) 7:20. doi: 10.20517/cdr.2024.25, PMID: 38835344 PMC11149099

[23] Toyokawa G Hirai F Inamasu E Yoshida T Nosaki K Takenaka T . Secondary mutations at I1171 in the ALK gene confer resistance to both Crizotinib and Alectinib. J Thorac Oncol. (2014) 9:e86–7. doi: 10.1097/JTO.0000000000000358, PMID: 25393798

[24] Shaw AT Friboulet L Leshchiner I Gainor JF Bergqvist S Brooun A . Resensitization to crizotinib by the lorlatinib ALK resistance mutation L1198F. N Engl J Med. (2016) 374:54–61. doi: 10.1056/NEJMoa1508887, PMID: 26698910 PMC4773904

[25] Katayama R Shaw AT Khan TM Mino-Kenudson M Solomon BJ Halmos B . Mechanisms of acquired crizotinib resistance in ALK-rearranged lung Cancers. Sci Transl Med. (2012) 4:120ra17. doi: 10.1126/scitranslmed.3003316, PMID: 22277784 PMC3385512

[26] Tang LB Peng YL Yang XR Li JT Lu C Zheng MM . Partial response to lorlatinib in thoracic inflammatory myofibroblastic tumor harboring complex and rare ALK fusions: a case report. Transl Lung Cancer Res. (2025) 14:631–8. doi: 10.21037/tlcr-24-963, PMID: 40114948 PMC11921266

